# Progress towards Healthy People 2030 population health goals

**DOI:** 10.1093/haschl/qxag026

**Published:** 2026-03-03

**Authors:** Siddharth Kesiraju, Shriya Garg, Urvish Jain, Aditya Arkalgud, Edward Christopher Dee, Joseph T Kannarkat, Sandro Galea

**Affiliations:** Dietrich School of Arts and Sciences, University of Pittsburgh, Pittsburgh, PA 15213, United States; Department of Economics, University of Georgia, Athens, GA 30602, United States; Dietrich School of Arts and Sciences, University of Pittsburgh, Pittsburgh, PA 15213, United States; Bennett School of Public Policy, University of Cambridge, Cambridge CB3 9DT, United Kingdom; Dietrich School of Arts and Sciences, University of Pittsburgh, Pittsburgh, PA 15213, United States; Department of Radiation Oncology, Memorial Sloan Kettering Cancer Center, New York, NY 10021, United States; Department of Medicine, Johns Hopkins University School of Medicine, Baltimore, MD 21224, United States; School of Public Health, Washington University in St Louis, St Louis, MI 63110, United States

**Keywords:** population health, public health, health equity, health policy, social determinants of health

## Abstract

**Objective:**

We aimed to quantify progress towards all 23 Healthy People 2030 leading health indicators (LHIs) national set targets. Understanding progress on these LHIs can guide policy reform that aims to improve US population health.

**Methods:**

Cross-sectional analysis based on de-identified, population-level federal data representing the US population, stratified by race, age, gender, income, and education using cluster-based and stratified sampling methods.

**Results:**

Of 23 LHIs, 5 were categorized as little to no detectable change, 5 as worsening, 6 as improving, 5 as target met or exceeded, and 2 with no updated current value. Notable improvements included treatment of adolescent depression, reducing binge drinking, and hypertension control, while worsening areas included drug-overdose deaths, homicides, and food insecurity. Inequalities have persisted or widened for most indicators.

**Conclusion:**

The United States is lagging or worsening in 10 of 23 Healthy People 2030 LHIs. Understanding the implications of such findings is valuable for highlighting health care gaps, pinpointing areas for improvement, and underscoring the need for action.

## Introduction

Healthy People 2030 (HP2030) is the latest iteration of a series of decennial initiatives by the US Department of Health and Human Services (HHS) to improve societal health and well-being, with set targets to be achieved by 2030.^1^ The initial iteration was introduced after the first published health report on health promotion and disease prevention by the Surgeon General in 1979. Since then, each iteration has built on the prior initiatives using new knowledge and lessons learned with priority areas of each decade's initiative slowly evolving from an emphasis on disease prevention and mortality reduction to a more holistic model, one that includes environmental, social, and behavioral determinants of health and an emphasis on health literacy and health equity.

Despite the United States spending more on health care per capita than do its peers, it has performed poorly in overall health care system performance and key health outcomes.^2^ Given this context, a centralized, coordinated, evidence-based framework such as HP2030 is essential in aligning key priorities, tracking progress, and guiding the action across relevant stakeholders. Achieving HP2030's emphasis on prevention, social determinants of health (SDOH), and health equity has the potential to improve overall population health while also bending the cost curve through reducing preventable health issues, which contribute to high health care spending.

Healthy People 2030 builds on previous initiatives by narrowing its focus on health equity and the social determinants of health. Introduced in August 2020, HP2030 has 3 main foci: health equity, health literacy, and SDOH such as income, education, neighborhood safety, and insurance coverage.^3^ Healthy People 2030 comprises 509 national objectives, 23 of which are categorized as leading health indicators (LHIs)—core objectives that track major causes of death and disease in the United States and serve as important benchmarks when assessing the nation's health.^4,5^ Compared with previous iterations, traditional LHIs focused on downstream outcomes, such as homicides, drug overdoses, and suicide, are still present in HP2030; however, many LHIs have been redesigned to place greater emphasis on upstream determinants of health, such as employment, education, environmental factors, and food insecurity.

There is a body of literature that has evaluated the conceptual frameworks of Healthy People initiatives, with some assessing progress toward goals and objectives in earlier iterations such as HP2010 and HP2020. These analyses have shown mixed results, with evaluations of HP2010 finding minimal improvement while lagging in overall goals and the HP2020 report highlighting a lack of progress or worsening in over 50% of LHIs.^6,7^ To date, there has been very little peer-reviewed work on the progress toward HP2030 targets, particularly for the LHIs.

In this study, we provide, to our knowledge, the first assessment of progress toward HP2030 LHI targets to determine if, based on current trajectories, these LHIs are on track to meet their HP2030 targets. This article complements existing analyses conducted since HP2010 and aims to illuminate new progress since such analyses have been made.^6^ These findings can inform prioritization and resource allocation to achieve HP2030 LHI targets.

## Data and methods

### Data sources

The Healthy People initiative is overseen by the Office of Disease Prevention and Health Promotion (ODPHP)—a division of the HHS. The Centers for Disease Control and Prevention’s (CDC's) National Center for Health Statistics (NCHS) works with ODPHP to maintain, update, and publish indicator values on the publicly accessible HP2030 website.

All HP2030 objectives contain data that were collected from more than 80 administrative records and national surveys. Each LHI is linked to a primary federal data system which may be either a large health survey (eg, National Youth Tobacco Survey),^8,9^ socioeconomic survey (eg, Current Population Survey Annual Social and Economic Supplement Census),^10^ or environmental monitoring network (eg, Environmental Protection Agency [EPA] Air Quality System [AQS] repository).^11^ The CDC/NCHS, US Department of Agriculture (USDA), EPA, Substance Abuse and Mental Health Services Administration (SAMHSA), and the US Census Bureau manage these systems. The frequency of LHI update status is based on the periodicity of each individual federal database update. Due to variability in source criteria as a result of combining datasets, the NCHS applies sampling weights and age-adjustment using the 2000 US Standard population.^12^

### Selection criteria

The LHIs were chosen for this analysis as they are a “subset of high priority HP2030 objectives” and “address important factors that impact major causes of diseases and death.”^5^ The LHIs were determined by the CDC based on recommendations from panels across the National Academies of Sciences, Engineering, and Medicine, a Federal Interagency Workgroup, and the HHS Secretary's Advisory Committee on National Health.^13-15^ Each LHI has an official objective code from HP2030 (eg, MHMD-06). These are codes associated with workgroups and topic areas. For organizational purposes, we created 4 thematic areas to group LHIs: Equitable Access and Social Determinants of Health, Behavioral Health Risk and Harm, Preventive and Chronic Disease Management, and Maternal and Infant Health. A comprehensive list of all LHIs examined within this study is available in Appendix Table S1.

### Progress analysis

Baseline, recent, and target values for each LHI were obtained from the HP2030 website. When more recent data were available for any LHI in the respective underlying federal data system, we retrieved and utilized those values using the same indicator definition. Data obtained via the HP2030 website were reported in 2022 or after. In the case of an LHI reporting metrics preceding 2022, the more recent data were extracted from the corresponding federal system database, if available. The LHIs for which the most recent available data were published before 2022 were as follows: reduce consumption of added sugars by people aged 2 years and over (NWS10), reduce the number of days people are exposed to unhealthy air (EH01). The underlying federal data systems for these LHIs are the National Health and Nutrition Examination Survey (NHANES) and the AQS, respectively. Next, progress toward each HP2030 target was assessed using the NCHS methodology.^16^ As data collection of each LHI varies in terms of methodology, progress was standardized through computation of a direction-corrected percentage change from baseline (PCB) for all LHIs and the percentage of targeted change achieved (PTCA) was computed for LHIs improving or meeting their target. The PTCA was calculated as follows: ([most recent value – baseline value]/[HP2030 target – baseline value]) × 100. The PCB was calculated using the following formula: ([most recent value – baseline value]/[baseline value]) × 100.

Healthy People 2030 has categorized each LHI as either improving, getting worse, little or no detectable change, target met or exceeded, or baseline only through quantitative assessment. All LHIs categorized as improving or worsening are statistically significant per the official NCHS methodology. The NCHS deemed statistical significance for each LHI using 1-sided tests at α = 0.05 if variance estimates were available, and PCB greater than 10% in either direction served as a proxy threshold for significance. Indicators were categorized as target met/exceeded if the 2030 target had been reached and little or no detectable change when either insignificant change or movement was less than 10% when measures of variability were unavailable.^12^ All analyses used Python version 3.12, and Microsoft Excel 2021 was used for descriptive summaries.

## Results

**Figure 1.**
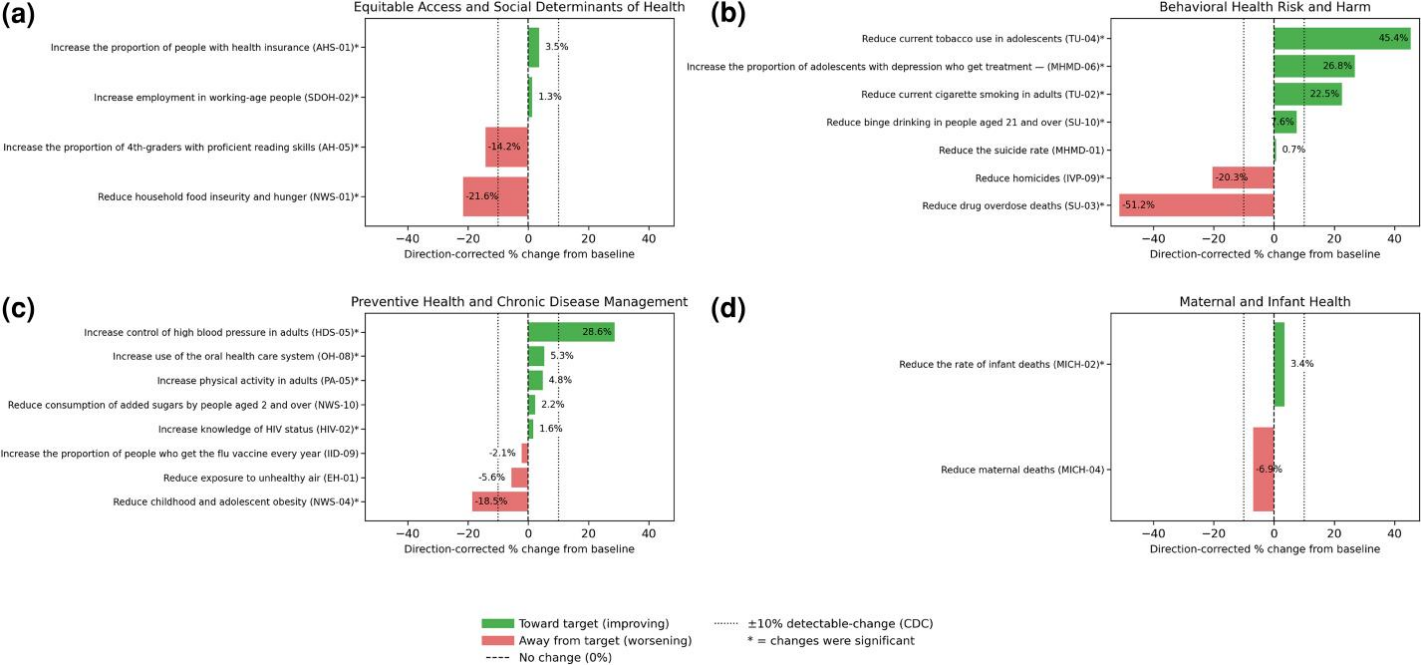
Direction-corrected percentage change from baseline for Healthy People 2030 leading health indicators (LHIs) organized by domain. Source: Healthy People 2030, Office of Disease Prevention and Health Promotion, US Department of Health and Human Services. Targets were established by federal subject-matter experts (SMEs) convened in HP2030 workgroups that include statisticians and epidemiologists from federal agencies.^4^ Percentage change from baseline of each LHI used the following formula: (current – baseline)/(baseline) × 100%. Abbreviation: CDC, Centers for Disease Control and Prevention.

**Figure 2.**
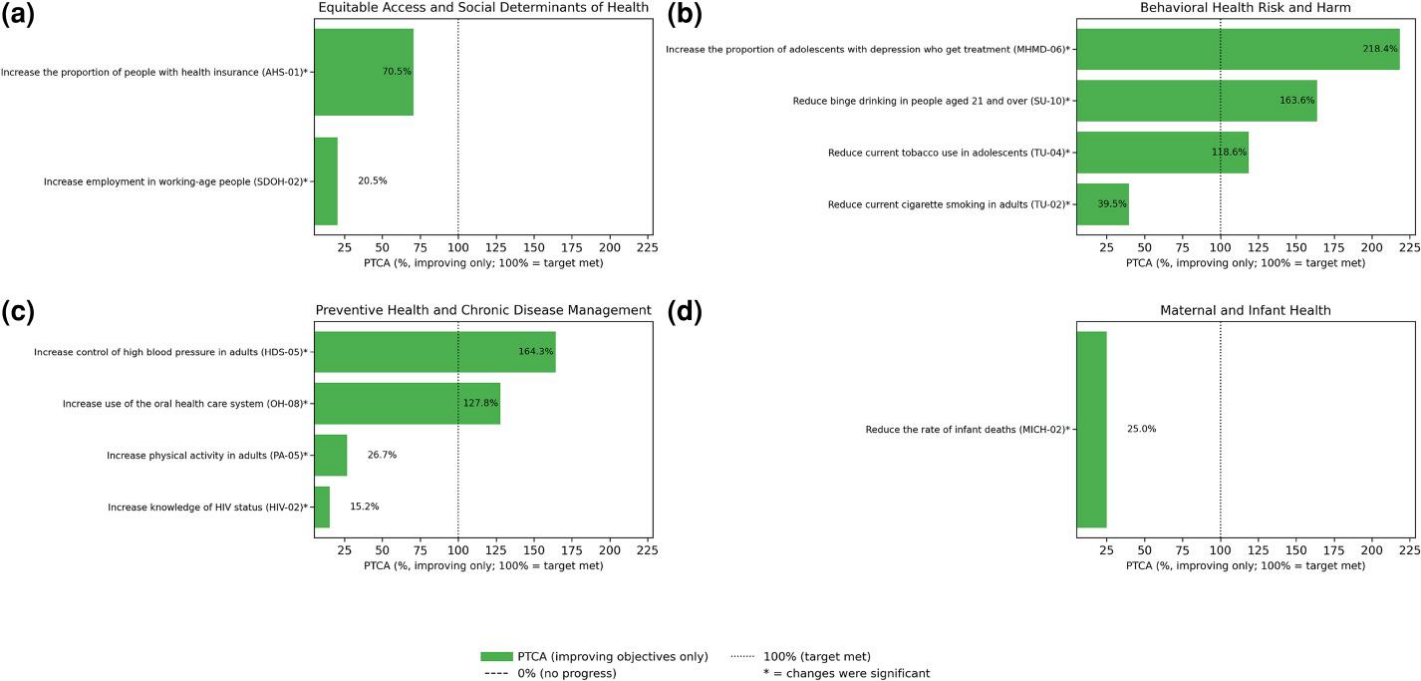
Percentage of targeted change achieved for Healthy People 2030 improving leading health indicators (LHIs) organized by domain. Source: Healthy People 2030, Office of Disease Prevention and Health Promotion, US Department of Health and Human Services. Targets were established by federal subject-matter experts (SMEs) convened in HP2030 workgroups that include statisticians and epidemiologists from federal agencies.^4^ Percentage change from baseline of each LHI used the following formula: (current – baseline)/(baseline) × 100%.

Of 23 LHIs, 5 were categorized as little to no detectable change, 5 as worsening, 6 as improving, 5 as target met or exceeded, and 2 as no updated current value (Figure 1). For the 11 that improved or met targets, the PCB and PTCA are shown (Figure 2). For all other LHIs, only the PCB is shown. Baseline-only LHIs include D-01 (reduce the number of diabetes cases diagnosed yearly) and C-07 (increase the proportion of adults who get screened for colorectal cancer).

### Equitable Access and Social Determinants of Health

Of the 4 Equitable Access and SDOH LHIs, 2 have improved and 2 have worsened (Appendix Table S2). The LHIs that improved include AHS-01 (increase the proportion of people with health insurance; PCB: 3.5%; PTCA: 70.5%) and SDOH-02 (increase employment in working-age people; 1.3%, 20.5%). The LHIs that have worsened include AH-05 (increase the proportion of fourth-graders with proficient reading skills; PCB: −15.0%) (Appendix Table S3) and NWS-01 (reduce household food insecurity and hunger; −21.1%) (Appendix Table S4).

### Behavioral Health Risks and Harm

Of 7 LHIs in this category (Appendix Table S5), 4 have improved, 2 have worsened, and 1 has not significantly changed. The LHIs that improved include MHMD-06 (increase the proportion of adolescents with depression who get treatment; PCB: 26.8%; PTCA: 218.4%), TU-02 (reduce current cigarette smoking in adults; 22.5%; 39.5%), TU-04 (reduce current tobacco use in adolescents; 45.4%; 118.6%), and SU-10 (reduce binge drinking in people aged 21 and over; 7.6%; 163.6%). The LHIs that worsened include IVP-09 (reduce homicides; PCB:−20.3%) (Appendix Table S6) and SU-03 (reduce drug-overdose deaths; −51.2%) (Appendix Table S7). The LHI with little or no significant change is MHMD-01 (reduce the suicide rate; 0.1%) (Appendix Table S8).

### Preventive Health and Chronic Disease Management

Of 8 LHIs in this category (Appendix Table S9), 4 have improved, 1 has worsened, and 3 have little to no detectable change. The LHIs that improved include OH-08 (increase use of the oral health care system; PCB: 5.3%; PTCA: 127.8%), PA-05 (increase physical activity among adults; 4.8%; 26.7%), HDS-05 (increase control of high blood pressure; 26.8%; 164.3%), and HIV-02 (increase knowledge of HIV status; 1.6%; 15.2%). The LHIs that have worsened include NWS-04 (reduce childhood and adolescent obesity; PCB: −18.5%). The LHIs that have little to no detectable change include NWS-10 (reduce consumption of added sugars by people aged 2 and over; 2.2%), IID-09 (increase the proportion of people who get the flu vaccine every year; −2.1%) (Appendix Table S10), and EH-01 (reduce exposure to unhealthy air; −5.6%).

### Maternal and Infant Health

Of the 2 LHIs in this category (Appendix Table S11), MICH-02 (reduce the rate of infant deaths; PCB: 3.3%; PTCA: 23.8%) has improved. The LHI with little to detectable change is MICH-04 (reduce maternal deaths; PCB: −6.9%) (Appendix Table S12).

## Discussion

At the midpoint of the HP2030 decade, this study seeks to evaluate the United States' progress towards its leading health indicators, revealing an uneven trajectory. Analyzing targets set for Equitable Access and SDOH, Behavioral Health Risks and Harm, Preventive Health and Chronic Disease Management, and Maternal and Infant Health, we highlight notable progress in certain healthy behaviors, including reducing cigarette and tobacco use, improving oral health, and managing blood pressure. Yet, limited progress and retreats in domains existed, with increases in homicide, suicide, and overdose rates and restrained improvement in food literacy and employment metrics. Further, while we are documenting progress towards specific targets, achievement of actual population health improvement may depend both on these metrics and by other, less easily measurable factors. For example, while health insurance coverage has increased, this is not accounting for quality and cost of coverage. Similarly, while employment for working-age people has improved, inflation and concomitant unaffordability of basic goods may be mitigating downstream effects of such progress.

These findings highlight a central issue with progress on HP2030: while national progress on several fronts is possible through coordinated efforts, progress towards improved population health remains hindered by a range of factors that are deeply linked to longstanding socioeconomic inequities that make progress unevenly distributed across specific population groups and demographic subsets.

### Equitable Access and Social Determinants of Health

Improving equitable access and SDOH are a major overarching goal under the HP2030 initiative. Areas where progress has been made include an improvement to 71.5% employment of working-age people since 2018 and 91.1% in health insurance coverage in the United States. This improvement in health insurance coverage expands affordable options for patients to see medical providers when needed, a benefit for population health.^17^

On the other hand, since 2010, the United States has continued to fall behind several HP2030 targets, such as in rates of food insecurity.^18,19^ In addition to higher rates of obesity, heart disease, and mental health issues, certain populations remain even more affected by such food insecurity—including mothers and children, who face significantly worse outcomes from pregnancy to age 2 years.^20^ Such outcomes may be attributed to the consumption of low-cost, highly processed foods, which can be more palatable than healthier alternatives. According to the CDC, between August 2021 and August 2023, the mean percentage of total calories consumed from ultra-processed foods among those above the age of 1 year was 55.0%, with youth between the ages of 1 and 18 years consuming higher percentages (61.9%) than adults older than 19 years (53.0%). Likewise, those in the highest family income group were found to have the lowest consumption of ultra-processed foods.^21^

Ongoing evidence-based interventions exist to reduce household food insecurity, such as Supplemental Nutritional Assistance Programs (SNAP), National School Lunch Program (NSLP), School Breakfast Program (SBP), and Food Banks and Pantries. However, implementation of these programs faces many challenges, including funding uncertainty, lack of program awareness for eligible individuals, complex application procedures, and limited efforts to address underlying SDOH.^22^ For example, the federally funded Special Supplemental Nutrition Program for Women, Infants, and Children (WIC) has enrolled just over half of the eligible applicants (51.2%).^23^ Such low uptake is additionally characterized by rural and urban divides, where people living in metropolitan areas (56%) are 2 times more likely to participate in the program compared with those living in nonmetropolitan areas (23%).^23^ Many WIC program administrators have witnessed an increase in participation alongside the promotion of these resources via social media, text messaging, and electronic modalities as well as through the streamlining of applications.^23^

The United States has also worsened in attempts to increase literacy among elementary-school-age students.^24,25^ In particular, the proportion of fourth-graders with proficient reading skills has significantly declined. This LHI is of particular interest as educational attainment has been linked to better health outcomes, employment, and health-promoting behaviors.^26,27^ In 2017, Black fourth-graders were approximately 3 times more likely to experience a deficiency in reading proficiency compared with Asian students and the latest 2023 data show the largest inequality in this group has worsened with American Indian/Alaska Native individuals, who are3.8 times more likely to experience a deficiency compared with Asian students, an indication of a widening inequality among underrepresented groups.^28^ It is important to note that prior research has shown significant heterogeneity in academic outcomes between Asian-American subgroups; however, these outcomes may become obscured when subgroups are grouped into a single category.^29^ Since these groups of students are more susceptible to negative outcomes associated with poor literacy rates, resources should be directed toward closing this gap. Initiatives such as enhanced teacher preparation programs, which include science of reading instruction for licensure, or better professional development courses on reading instruction practices could help ensure that teachers are well prepared to identify children demonstrating signs of illiteracy and help support them.^30^ The decline in literacy rates may reflect educational system lay-offs and lengthy school closures attributed to the COVID-19 pandemic.^31^

### Behavioral Health Risks and Harms

Three of 7 LHIs (reducing the suicide rate, reducing drug-overdose deaths, reducing homicides) in this category were classified as having little or no detectable change or worsening. The United States has made efforts to increase the proportion of adolescents who get treated for depression as major depressive disorder is associated with a higher risk of suicide.^32^ However, despite such efforts, the suicide rate has remained stagnant, and the drug-overdose rate has worsened. One possible explanation may be due to overdiagnosis and overtreatment of depression.^33^ Moreover, HP2030 data show that males are 3.7 times more likely to commit suicide than females, a persistent trend that may reflect a lack of help-seeking behavior and method of lethality as men are less likely to access mental health services and more likely to use firearms.^34-36^

While the drug-overdose rate is still higher than 2020 levels, a recent CDC release showed that drug-overdose deaths have declined by almost 25% in the 12 months ending in September 2024.^37^ The CDC also notes that drug overdoses are the leading cause of death for individuals aged 18–44 years, reemphasizing the need for close monitoring of this LHI to ensure sustained progress. Additionally, data have shown that males disproportionately experienced more deaths than females from drug overdoses and suicides, illustrating a need for targeted interventions in male populations.^5,38^ While the overall age-adjusted rate of drug-overdose deaths decreased by 4.0% between 2022 and 2023, increased emphasis on programming to educate about the harms of drug use (particularly those outlined by the National Institute on Drug Abuse), greater dissemination of resources such as Narcan that prevent opioid overdose, and decreased stigma that deters users from seeking out help are important to combatting this issue.^39-42^

Worsening progress in reducing homicides (IVP-09) remains a concern.^43^ While homicide levels are still elevated from the beginning of the decade, there was a significant surge during the COVID-19 pandemic, with homicide levels consistently declining since peaking in late 2022.^43^ In 2020, 77% of murders in the United States were committed by firearms, while simultaneously the United States experienced a surge in firearm purchases.^44,45^ Furthermore, changes in law enforcement, such as a 279% increase in police officer resignations in the second half of 2020, in addition to mass unemployment, school closures, and societal disruption, may have contributed to risks of violence.^46,47^

### Preventive Health and Chronic Disease Management

While shortcomings persist, much progress has been made in preventive health and chronic disease management. Notably, the United States has increased the use of the oral health care system (OH-08), which has met the HP2030 target as recently as 2022. Such improvements are likely the result of a multitude of changes. First, the integration of oral health into larger health delivery systems and primary care practices has likely helped to prioritize oral health for patients.^48,49^ Further, the increase in tele-dentistry has helped to overcome low provider densities, financial barriers, travel and wait times, and allow for increased preventive care approaches.^50-52^ The National Rural Health Association cites that, since 2020, more than 85 000 virtual dental health visits have been conducted for triage, postoperative follow-up, and exams.^53^ Last, the increase in patient navigators, such as community dental health coordinators, has helped to address the need for oral care in low-income or underserved populations.^54^ Frequent dental visits can enable earlier detection of oral cancer, gum disease, and cavities for proactive treatment, which will improve the overall health of residents of the United States.^55^ Still, significant improvements can be made through the continued emphasis on the fluoridation of water, the deregulation of direct access for dental hygienists, and the expansion of dental care coverage.^56^

Although the United States has progressed in overall physical activity among adults, progress in preventive and chronic disease management strategies for older populations has lagged, exemplified by the failure of older US adults (45–64 years) to meet the aerobic and muscle-strengthening activity guideline target. However, on a population level, physical activity has improved since 2020.^57^ On the other hand, individuals aged 18–44 years have exceeded the aerobic and muscle strengthening (PA-05) activity guideline overall target of 29.7%.

Despite the progress made in oral health and physical activity, childhood obesity (NWS-04) remains a pressing issue for the United States, with 57.3% of US children predicted to be obese by 2050.^58,59^ Childhood obesity has been linked to a 3-times higher mortality rate before the age of 30; increased risk of cardiovascular disease, diabetes, and certain cancers; and increased psychological comorbidities.^60^ Furthermore, a key driver of rising rates of childhood obesity is the obesogenic environment in which children are brought up.^61,62^

Last, although the United States has not significantly regressed in exposure to unhealthy air, HP2030 estimates that 100 000–200 000 deaths annually are due to poor quality of air within the United States. Such estimates do not account for the poor life quality as a result of poor air quality, which is associated with increased health problems such as lung cancer and heart disease.^63^

### Maternal and Infant Health

Although there has been progress in reducing infant deaths, maternal deaths have slightly risen. In 2018, there were 17.4 maternal deaths per 100 000 live births and this number has increased to 18.6 in 2023.^64,65^ While approximately 80% of maternal death cases are preventable, the United States still struggles to lower its maternal mortality rate and has approximately twice the rate of maternal deaths in 2020 compared with other high-income countries.^66^ There are also notable inequalities, with Black mothers dying at a 3.5-times higher rate than White mothers and 4.7 times more than Asian mothers.^65^ This stems from factors such as implicit bias in clinical settings, economic disadvantage, limited access to quality services, and structural factors.^67^

Although maternal deaths have increased overall, maternal mortality specifically during delivery and labor has decreased in the United States.^68^ This indicates that inadequate postpartum care may be a cause of such high mortality burdens.^69^ Such findings are supported by data that show that more than 30% of pregnancy-related maternal deaths are between 6 weeks and 1 year after childbirth, with mental health conditions such as postpartum depression contributing to 22.7% of deaths.^68^

### Strengths and limitations

In terms of strengths, the study used standardized, nationally representative data provided by the ODPHP. The national surveys and administrative records, such as National Health and Nutrition Examination Survey, National Health Interview Survey, Behavioral Risk Factor Surveillance System, and the US Census Bureau, collect and validate their data allowing for analysis that is transparent and reproducible.

However, several limitations exist. Since this study is based on LHIs only and not the full complement of HP2030 objectives, the results may not be generalizable. Data for each LHI are derived from a range of sources, collected using various sample methods and survey design, and the frequency of data collection also varies. Variation in data methods can affect the accuracy of comparability of LHIs. Additionally, the year of baseline values and recent value measurement vary across LHIs, making it difficult to accurately compare progress achievement across LHIs at a specific moment in time. Further, most LHIs rely on self-reported data (eg, NHIS and BRFSS). This can result in a reporting bias, desirability bias, and even error in recall. Another limitation of the study is that the frequency of data collection varies among LHIs, and the lack of annual data collection makes it difficult to track and respond to changes in the short term. Moreover, groupings of LHIs into categories is intended to clarify and organize the analyses presented and should not be used for intergroup comparisons.

Moreover, the recent dissolution of several federal health databases may make future tracking for HP outcomes difficult and unstandardized. Finally, the generalizability of some of the findings may be limited as there may be a response bias present. Additionally, data lags may impact policy action and evaluation timing.

## Conclusion

Our analysis reveals mixed results on the progress toward HP2030 goals. In 2025, 10 of 23 LHIs have either worsened or shown little to no detectable change. These LHIs include major primary endpoints such as reducing suicides, homicides, household food insecurity, and drug-overdose deaths.

On the other hand, significant progress has been made in other areas that align with HP2030's aim to promote healthy behaviors, expand access to preventive and clinical services, and foster healthy development across life stages. Notable LHIs include increasing hypertension control, use of the oral health system, physical activity, health insurance coverage, adolescent depression treatment, and reducing binge drinking among those aged 21 years.

The progress of LHIs under HP2030 mirrors past iterations such as HP2020. In prior initiatives, meaningful progress was made in objectives pertaining to behavior or clinical care and fewer improvements were made in objectives categorized into SDOH. The HP2020 end-of-decade snapshot revealed worsening trends in substance use, mental health, obesity, and health inequalities. Many of these worsening trends have intensified under the current initiative. Despite improvements in clinical outcomes, other important endpoints of health such as violence, poverty, and food insecurity have been resistant to current intervention strategies. Multifaceted action from policymakers, public health officials, communities, educators, and innovators is needed to help meet HP2030 goals, particularly those upstream of health pertaining to premature death and disability.

## Supplementary Material

qxag026_Supplementary_Data

## Data Availability

Data is available publically at https://odphp.health.gov/healthypeople.

## References

[1] Mager ND, Moore TS. Healthy People 2030: roadmap for public health for the next decade. Am J Pharm Educ. 2020;84(11):8462. 10.5688/ajpe8462 34283761 PMC7712733

[2] Commonwealth Fund . Health Care by Country 2024 Report. Accessed January 9, 2026. https://www.commonwealthfund.org/publications/fund-reports/2024/sep/mirror-mirror-2024

[3] Spruce L . Back to basics: social determinants of health. AORN J. 2019;110(1):60–69. 10.1002/aorn.12722 31246307

[4] Hubbard K, Talih M, Klein R. Targetsetting Methods in Healthy People 2030. Healthy People Statistical Notes, No 28. Updated 2020. 2025. https://www.cdc.gov/nchs/data/statnt/statnt28-508.pdf

[5] odphp.health.gov. Leading health indicators—Healthy People 2030. Accessed May 17, 2025. https://odphp.health.gov/healthypeople/objectives-and-data/leading-health-indicators

[6] Sondik EJ, Huang DT, Klein RJ, Satcher D. Progress toward the Healthy People 2010 goals and objectives. Annu Rev Public Health. 2010;31(1):271–281. 10.1146/annurev.publhealth.012809.103613 20070194

[7] Koh HK, Blakey CR, Roper AY. Healthy People 2020: a report card on the health of the nation. JAMA. 2014;311(24):2475–2476. 10.1001/jama.2014.6446 24870206

[8] odphp.health.gov. Reduce consumption of added sugars by people aged 2 years and over—Data Methodology and Measurement—Healthy People 2030. Accessed May 17, 2025. https://odphp.health.gov/healthypeople/objectives-and-data/browse-objectives/nutrition-and-healthy-eating/reduce-consumption-added-sugars-people-aged-2-years-and-over-nws-10/data-methodology

[9] Centers for Disease Control and Prevention (CDC) . About National Youth Tobacco Survey (NYTS). Smoking and tobacco use. Updated May 3, 2024. Accessed May 17, 2025. https://www.cdc.gov/tobacco/about-data/surveys/national-youth-tobacco-survey.html

[10] odphp.health.gov. National Vital Statistics System—Mortality (NVSS-M)—Healthy People 2030. Accessed May 17, 2025. https://odphp.health.gov/healthypeople/objectives-and-data/data-sources-and-methods/data-sources/national-vital-statistics-system-mortality-nvss-m

[11] odphp.health.gov. Air Quality System (AQS)—Healthy People 2030. Accessed May 17, 2025. https://odphp.health.gov/healthypeople/objectives-and-data/data-sources-and-methods/data-sources/air-quality-system-aqs

[12] Huang DT, Uribe A, Makram T. Measuring progress toward target attainment and the elimination of health disparities in Healthy People 2030: data evaluation and methods research. Accessed May 17, 2025. https://stacks.cdc.gov/view/cdc/164019 10.15620/cdc/164019 PMC12278040 40576483

[13] National Health Initiatives, Office of Disease Prevention and Health Promotion . odphp.health.gov. Accessed January 9, 2026. https://odphp.health.gov/our-work/national-health-initiatives/healthy-people/healthy-people-2030/federal-interagency-workgroup

[14] National Academies of Sciences, Engineering, and Medicine . Informing the selection of leading health indicators for Healthy People 2030. Accessed January 9, 2026. https://www.nationalacademies.org/projects/HMD-BPH-18-16

[15] Secretary's Advisory Committee on National Disease Health Promotion and Disease Prevention Objectives for 2030: Recommendations for the Healthy People 2030 Leading Health Indicators. January 9, 2026. https://odphp.health.gov/sites/default/files/2021-09/Committee-LHI-Report-to-Secretary_1.pdf

[16] Huang D, Uribe A, Makram T. Measuring Progress Toward Target Attainment and the Elimination of Health Disparities in Healthy People 2030. National Center for Health Statistics (U.S.); 2024. 10.15620/cdc/164019 PMC12278040 40576483

[17] Ercia A . The impact of the Affordable Care Act on patient coverage and access to care: perspectives from FQHC administrators in Arizona, California and Texas. BMC Health Serv Res. 2021;21(1):920. 10.1186/s12913-021-06961-9 34488758 PMC8420058

[18] Gucciardi E, Vahabi M, Norris N, Del Monte JP, Farnum C. The intersection between food insecurity and diabetes: a review. Curr Nutr Rep. 2014;3(4):324–332. 10.1007/s13668-014-0104-4 25383254 PMC4218969

[19] Ejiohuo O, Onyeaka H, Unegbu KC, et al Nourishing the mind: how food security influences mental wellbeing. Nutrients. 2024;16(4):501. 10.3390/nu16040501 38398825 PMC10893396

[20] Bell Z, Nguyen G, Andreae G, et al Associations between food insecurity in high-income countries and pregnancy outcomes: a systematic review and meta-analysis. PLoS Med. 2024;21(9):e1004450. 10.1371/journal.pmed.1004450 39255262 PMC11386426

[21] Williams A, Couch C, Emmerich S, Ogburn D. Products—Data Briefs—Number 536—August 2025. https://stacks.cdc.gov/view/cdc/174612 10.15620/cdc/174612 PMC12516466 41027400

[22] Rudolph C, Francis S. Making home-delivered meal programs relevant for today’s aging adult. J Public Health (Berl.). 2022;30:141–150. 10.1007/s10389-020-01286-z

[23] Center on Budget and Policy Priorities. WIC’s critical benefits reach only half of those eligible. Updated February 21, 2024. Accessed January 9, 2026. https://www.cbpp.org/research/food-assistance/wics-critical-benefits-reach-only-half-of-those-eligible

[24] Park H, Kyei P. Literacy gaps by educational attainment: a cross-national analysis. Soc Forces. 2011;89(3):879–904. 10.1353/sof.2011.0025 21818163 PMC3148089

[25] Kuhfeld M, Lewis K, Peltier T. Reading achievement declines during the COVID-19 pandemic: evidence from 5 million U.S. students in grades 3-8. Read Writ. 2023;36(2):245–261. 10.1007/s11145-022-10345-8 35991159 PMC9383679

[26] Organization for Economic Cooperation and Development (OECD) . How does educational attainment affect participation in the labour market? Education at a Glance 2025. Updated September 9, 2025. Accessed January 9, 2026. https://www.oecd.org/en/publications/education-at-a-glance-2025_1c0d9c79-en/full-report/how-does-educational-attainment-affect-participation-in-the-labour-market_ae451464.html

[27] Zajacova A, Lawrence EM. The relationship between education and health: reducing disparities through a contextual approach. Annu Rev Public Health. 2018;39(1):273–289. 10.1146/annurev-publhealth-031816-044628 29328865 PMC5880718

[28] odphp.health.gov. Increase the proportion of 4^th^-graders with reading skills at or above the proficient level—Data—Healthy People 2030. Accessed January 9, 2026. https://odphp.health.gov/healthypeople/objectives-and-data/browse-objectives/schools/increase-proportion-4th-graders-reading-skills-or-above-proficient-level-ah-05/data?group=Race%2FEthnicity&state=United States

[29] Pang VO, Han PP, Pang JM. Asian American and Pacific Islander students: equity and the achievement gap. Educ Res. 2011;40(8):378–389. 10.3102/0013189X11424222

[30] Center for American Progress . Improving literacy in the United States: recommendations for increasing reading success. Updated May 28, 2020. Accessed January 9, 2026. https://www.americanprogress.org/article/improving-literacy-united-states-recommendations-increasing-reading-success/

[31] Azevedo JP, Hasan A, Goldemberg D, Geven K, Iqbal SA. Simulating the potential impacts of COVID-19 school closures on schooling and learning outcomes: a set of global estimates. World Bank Res Obs. 2021:lkab003. 10.1093/wbro/lkab003

[32] Brådvik L . Suicide risk and mental disorders. Int J Environ Res Public Health. 2018;15(9):2028. 10.3390/ijerph15092028 30227658 PMC6165520

[33] Mojtabai R . Clinician-identified depression in community settings: concordance with structured-interview diagnoses. Psychother Psychosom. 2013;82(3):161–169. 10.1159/000345968 23548817

[34] odphp.health.gov. Reduce the suicide rate—Data—Healthy People 2030. Accessed January 9, 2026. https://odphp.health.gov/healthypeople/objectives-and-data/browse-objectives/mental-health-and-mental-disorders/reduce-suicide-rate-mhmd-01/data?group=Sex&state=United States

[35] Siegel M, Rothman EF. Firearm ownership and suicide rates among US men and women, 1981–2013. Am J Public Health. 2016;106(7):1316–1322. 10.2105/AJPH.2016.303182 27196643 PMC4984734

[36] Bennett S, Robb KA, O’Connor RC. Male suicide and barriers to accessing professional support: a qualitative thematic analysis. Curr Psychol. 2024;43(17):15125–15145. 10.1007/s12144-023-05423-1

[37] Centers for Disease Control and Prevention (CDC) . CDC reports nearly 24% decline in U.S. drug overdose deaths. CDC Newsroom. Updated February 25, 2025. Accessed May 17, 2025. https://www.cdc.gov/media/releases/2025/2025-cdc-reports-decline-in-us-drug-overdose-deaths.html

[38] Butelman ER, Huang Y, Epstein DH, et al Overdose mortality rates for opioids and stimulant drugs are substantially higher in men than in women: state-level analysis. Neuropsychopharmacology. 2023;48(11):1639–1647. 10.1038/s41386-023-01601-8 37316576 PMC10517130

[39] López-Ramírez E, Huber MJ, Matías-Pérez D, Santos-López G, García-Montalvo IA. Opioid harm reduction and stigma: proposed methods to improve the perception of people with addiction. Front Psychiatry. 2023;14:1197305. 10.3389/fpsyt.2023.1197305 37636822 PMC10447975

[40] Centers for Disease Control and Prevention (CDC) . Preventing opioid overdose. Overdose prevention. Updated August 12, 2025. Accessed October 4, 2025. https://www.cdc.gov/overdose-prevention/prevention/index.html

[41] Department of Health and Human Services . US Surgeon General’s advisory on naloxone and opioid overdose. Updated April 3, 2018. Accessed January 9, 2026. https://www.hhs.gov/surgeongeneral/reports-and-publications/addiction-and-substance-misuse/advisory-on-naloxone/index.html

[42] National Institute on Drug Abuse . Lesson plans and activities. Accessed January 9, 2026. https://nida.nih.gov/research-topics/parents-educators/lesson-plans-and-activities

[43] Federal Bureau of Investigation Crime Data Explorer . Accessed January 9 2026. https://cde.ucr.cjis.gov/latest/webapp/

[44] Gramlich J . What we know about the increase in U.S. murders in 2020. Pew Research Center. Updated October 27, 2021. Accessed May 17, 2025. https://www.pewresearch.org/short-reads/2021/10/27/what-we-know-about-the-increase-in-u-s-murders-in-2020/

[45] Federal Bureau of Investigation . NICS firearm checks: month/year. Accessed May 17, 2025. https://www.fbi.gov/file-repository/cjis/nics_firearm_checks_-_month_year.pdf/view

[46] Mourtgos SM, Adams IT, Nix J. Elevated police turnover following the summer of George Floyd protests: a synthetic control study. Criminol Public Policy. 2022;21(1):9–33. 10.1111/1745-9133.12556

[47] Brookings. Why did U.S. homicides spike in 2020 and then decline rapidly in 2023 and 2024? Accessed May 17, 2025. https://www.brookings.edu/articles/why-did-u-s-homicides-spike-in-2020-and-then-decline-rapidly-in-2023-and-2024/

[48] Prasad M, Manjunath C, Murthy K, et al Integration of oral health into primary health care: a systematic review. J Family Med Prim Care. 2019;8(6):1838–1845. 10.4103/jfmpc.jfmpc_286_19 31334142 PMC6618181

[49] Christian B, George A, Veginadu P, et al Strategies to integrate oral health into primary care: a systematic review. BMJ Open. 2023;13(7):e070622. 10.1136/bmjopen-2022-070622 PMC10367016 37407034

[50] Carter N . Teledentistry: a global solution with local impact. BDJ Pract. 2024;37(5):170–171. 10.1038/s41404-024-2724-8

[51] Islam MRR, Islam R, Ferdous S, et al Teledentistry as an effective tool for the communication improvement between dentists and patients: an overview. Healthcare. 2022;10(8):1586. 10.3390/healthcare10081586 36011243 PMC9408418

[52] Al-Buhaisi D, Karami S, Gomaa N. The role of teledentistry in improving oral health outcomes and access to dental care: an umbrella review. J Oral Rehabil. 2024;51(11):2375–2389. 10.1111/joor.13836 39138933

[53] National Rural Health Association . NRHA’s Rural Health Voices Blog. Accessed September 27, 2025. https://www.ruralhealth.us/blogs/2025/08/four-ways-rural-communities-are-improving-oral-health-equity

[54] Community Dental Health Coordinator . Accessed September 27, 2025. https://www.ada.org/resources/community-initiatives/action-for-dental-health/community-dental-health-coordinator

[55] Langevin SM, Michaud DS, Eliot M, Peters ES, McClean MD, Kelsey KT. Regular dental visits are associated with earlier stage at diagnosis for oral and pharyngeal cancer. Cancer Causes Control. 2012;23(11):1821–1829. 10.1007/s10552-012-0061-4 22961100 PMC3469776

[56] National Conference of State Legislatures. Dental hygienists with direct access—scope of practice policy. Accessed September 27, 2025. https://www.ncsl.org/scope-of-practice-policy/practitioners/oral-health-professionals/dental-hygienists-with-direct-access

[57] odphp.health.gov. Increase the proportion of adults who do enough aerobic and muscle-strengthening activity—Data—Healthy People 2030. Accessed May 17, 2025. https://odphp.health.gov/healthypeople/objectives-and-data/browse-objectives/physical-activity/increase-proportion-adults-who-do-enough-aerobic-and-muscle-strengthening-activity-pa-05/data

[58] Hills AP, Andersen LB, Byrne NM. Physical activity and obesity in children. Br J Sports Med. 2011;45(11):866–870. 10.1136/bjsports-2011-090199 21836171

[59] Ward ZJ, Long MW, Resch SC, Giles CM, Cradock AL, Gortmaker SL. Simulation of growth trajectories of childhood obesity into adulthood. N Engl J Med. 2017;377(22):2145–2153. 10.1056/NEJMoa1703860 29171811 PMC9036858

[60] Marcus C, Danielsson P, Hagman E. Pediatric obesity—long-term consequences and effect of weight loss. J Intern Med. 2022;292(6):870–891. 10.1111/joim.13547 35883220 PMC9805112

[61] Di Cesare M, Sorić M, Bovet P, et al The epidemiological burden of obesity in childhood: a worldwide epidemic requiring urgent action. BMC Med. 2019;17(1):212. 10.1186/s12916-019-1449-8 31760948 PMC6876113

[62] Lindberg L, Danielsson P, Persson M, Marcus C, Hagman E. Association of childhood obesity with risk of early all-cause and cause-specific mortality: a Swedish prospective cohort study. PLoS Med. 2020;17(3):e1003078. 10.1371/journal.pmed.1003078 32187177 PMC7080224

[63] World Health Organization. Ambient (outdoor) air pollution. Accessed May 17, 2025. https://www.who.int/news-room/fact-sheets/detail/ambient-(outdoor)-air-quality-and-health

[64] Hoyert DL, Miniño AM. Maternal mortality in the United States: changes in coding, publication, and data release, 2018. Natl Vital Stat Rep. 2020;69(2):1–18. 32510319

[65] Centers for Disease Control and Prevention. Health E-Stat 100: maternal mortality rates in the United States, 2023. Updated May 1, 2025. Accessed May 17, 2025. https://www.cdc.gov/nchs/data/hestat/maternal-mortality/2023/maternal-mortality-rates-2023.htm

[66] Fleszar LG, Bryant AS, Johnson CO, et al Trends in state-level maternal mortality by racial and ethnic group in the United States. JAMA. 2023;330(1):52–61. 10.1001/jama.2023.9043 37395772 PMC10318476

[67] Crear-Perry J, Correa-de-Araujo R, Lewis Johnson T, McLemore MR, Neilson E, Wallace M. Social and structural determinants of health inequities in maternal health. J Womens Health. 2021;30(2):230–235. 10.1089/jwh.2020.8882 PMC8020519 33181043

[68] Fink DA, Kilday D, Cao Z, et al Trends in maternal mortality and severe maternal morbidity during delivery-related hospitalizations in the United States, 2008 to 2021. JAMA Netw Open. 2023;6(6):e2317641. 10.1001/jamanetworkopen.2023.17641 37347486 PMC10288331

[69] Hippensteele A . Confronting the maternal mortality crisis in the United States: insights from the 2025 Commonwealth Fund Brief. AJMC. Updated January 9, 2026. Accessed January 9, 2026. https://www.ajmc.com/view/confronting-the-maternal-mortality-crisis-in-the-united-states-insights-from-the-2025-commonwealth-fund-brief

