# Supplementary material for: Progress towards Healthy People 2030 population health goals
Source: Health Aff Sch. 2026 Mar 3;4(3):qxag026. doi: 10.1093/haschl/qxag026 (PMC12955763; doi:10.1093/haschl/qxag026)
Supplement: qxag026_Supplementary_Data [file qxag026_supplementary_data.zip › HP2030_Appendix 01272026.docx]

**APPENDIX**

**Appendix Methods**

Disparities for LHIs categorized as worsening or little to no detectable change were assessed for notable demographic subgroup categories (e.g., sex, age, race/ethnicity, family income, geography). Publicly available data on the HP website was retrieved for these analyses. Calculations were done for both baseline year and recent year using two measures to capture maximal differences across population subgroups: maximal rate difference and maximal rate ratio. Maximal rate differences were the absolute differences between the best performing and lowest performing group. Maximal rate ratios were the ratio of the highest to lowest group rates. HP2030 uses a resampling/bootstrapping procedure to estimate the SEs and confidence intervals for all four measures. This procedure 1) draws 25,000 samples from a normal distribution of each group, 2) generates 25,000 estimates of rankings, reference rates, and disparity measures, and 3) computes the empirical SEs and confidence intervals using the interquartile range. All data are de-identified and aggregated to protect confidentiality.^11^

Significance of max differences and ratios were calculated using available standard errors (SE) and/or 95% confidence intervals (CIs) published by HP for each individual value. Statistical significance for differences was confirmed if CI excludes 0 and statistical significance for ratios was confirmed if CI excludes 1. If CIs were available, they were used, however when only SEs were provided, assuming normality, 95% CI of subgroup values were computed using the following formula: CI = X ± 1.96 x SE. For the computed differences and ratios, the difference SE and Ratio CI were both calculated. The 95% CI for the Maximal difference Ratio (MDR) was calculated by first individually squaring the SEs of both groups, then adding them together, and then taking the square root of the sum. This output could then be plugged into the following 95% CI formula: diff ± 1.96 x SEdiff. For Maximal Rate Ratios (MRR) the log scale was used. CI was computed by taking the respective SE of both groups and dividing each by their respective value, squaring each term individually, and taking the square root of the sum. This was then calculated using the following 95% CI formula: ln (MRR) ± 1.96 × SEln(MRR).

**Appendix Results**

*Equitable Access and SDOH*

NWS-01

2018 baseline data show that households with one or more disabled adults experience a higher rate of food insecurity compared to households with no disabled adults. The most recent 2023 data show that since the baseline year of 2018, the rate of food insecurity has increased among both groups. The absolute disparity between the two groups has remained around the same with a rate difference of 12.7 percentage points while the relative disparity gap has slightly narrowed with the rate ratio decreasing from 2.6 to 2.2.

When stratified by family income, 2018 baseline data show that the largest disparity gap was between households at <100% of the poverty threshold and households at 400%+ the poverty threshold, with the former experiencing a higher rate of food insecurity. The most recent data from 2023 shows that since 2018, the largest disparity gap has remained between these groups that both experience a higher rate of food insecurity. The absolute disparity gap has increased a maximal rate difference to 35.1 percentage points from 33.4 percentage points; however, the relative disparity gap has narrowed from a maximal rate ratio of 18.6 to 11.0.

AH-05

Data from 2017 show that 4^th^ graders eligible for the school lunch program experience a lower reading proficiency rate compared to students that are ineligible. The most recent data from 2022 show that since 2017, reading proficiency fell among both groups. The absolute disparity gap between these groups has slightly improved from a maximal rate difference of 29.8 percentage points to 27.1 percentage points, however the relative disparity gap has remained the same at a maximal rate ratio of 2.4.

Moreover, the largest racial reading proficiency gap in 2017 among 4^th^ graders was between Black or African American only and Asian only students, with black students experiencing a lower rate of reading proficiency. This has an absolute disparity gap of 38.8 percentage points and a relative disparity gap with a rate ratio of 3.0. The most recent data from 2024 show that since 2017, the largest gap in reading proficiency moved between American Indian/Alaska Native only and Asian only students, an absolute disparity gap which rose to 39.1 percentage points and an increase in relative disparity gap to a ratio of 3.8.

*Behavioral Health Risks and Harm*

SU-03

Baseline data from 2018 show the largest age gap in behavioral health between individuals aged 45-64 years and those <18 years, with the former experiencing a higher rate of drug overdoses. Most recent data from 2023 show this disparity gap persists and the drug overdose rate has increased for both groups. The absolute disparity gap between these groups has increased from a maximal rate difference of 31.2 deaths to 49.8 deaths while the relative disparity gap has dropped from a maximal rate ratio of 63.0 to 36.6.

Furthermore, baseline data from 2018 show that males experienced more deaths than females from drug overdose. Most recent data from 2023 show overall deaths have increased in both groups. Both the absolute disparity gap and the relative disparity gap widened with the maximal rate difference increasing from 14.3 deaths to 26.0 deaths and maximal rate ratio increasing from 2.1 to 2.4.

When stratified by geographic location, baseline data from 2018 show individuals in metropolitan areas experienced slightly more deaths from overdose compared to non-metropolitan areas. More recent 2021 data show the rate of drug overdoses has increased in both groups. The absolute disparity gap has narrowed from a maximal rate difference 2.0 deaths to 0.2 deaths and the relative disparity gap has remained around the same at 1.0.

MHMD-01

When stratified by sex, baseline data from 2018 show males committed a higher rate of suicides than females. Recent 2023 data show the rate of suicide among both groups has remained largely stagnant. The absolute disparity gap and relative disparity gap have also remained around the same with a maximal rate difference of 16.6 suicides and maximal rate ratio of 3.7.

Indeed, 2018 data show the largest age disparity gap was between individuals aged 45-64 years and those <18 years of age, with the former experiencing a higher rate of suicides. Recent 2023 data show that this disparity persists and the rate of suicides among those aged 45-64 years has decreased while the rate among those less than 18 years has remained stagnant. The absolute disparity gap has slightly decreased from a maximal rate difference of 17.6 suicides to 16.6 suicides; however, the relative disparity gap has slightly increased from a maximal rate ratio of 8.0 to 8.5.

IVP-09

2018 data show males committed homicides at a higher rate than their female counter part. Recent 2023 data show the homicide rate increased for males but remained stagnant for females. Both absolute disparity gap and relative disparity gap have increased from a maximal rate difference of 6.8 homicides to 8.5 homicides and a maximal rate ratio from 3.7 to 4.04.

In terms of age, 2018 data show the largest disparity gap was between individuals aged 18-44 years and individuals 65+ and <18 years, with the former committing a higher rate of homicides. Recent data from 2023 show that this disparity persists the rate of homicides has increased for those aged 18-44 while remained largely stagnant for individuals aged 65+ and under 18. The absolute disparity gap has increased from a maximal rate difference of 8.4 homicides to 10 homicides, and the relative disparity gap has increased from a maximal rate ratio of 4.8 to 5.3.

Geographically, 2018 data show metropolitan areas experience a higher rate of homicides than non-metropolitan areas. The latest data from 2021 show that the overall homicide rate increased in both locations. The absolute disparity gap has increased from a maximal rate difference of 0.5 homicides to 1 homicide, and the relative disparity gap has remained relatively stagnant at 1.14.

*Preventive Health and Chronic Disease Management*

IID-09

When stratified by health insurance status, baseline data from 2019-2020 data show that the largest disparity gap was between uninsured individuals and publicly insured individuals, with the former experiencing a lower rate of vaccinations — a maximal rate difference of 32.1 vaccinations and maximal rate ratio of 2.3. Recent 2022-2023 data show that the rate of flu vaccination has dropped across all groups and the largest disparity gap is now between uninsured individuals and privately insured individuals with a maximal rate difference of 31.1 vaccinations and maximal rate ratio of 2.4. Overall, the relative disparity gap between groups remains the same. Notably, vaccination status among those on public insurance compared to those on private insurance did not differ much.

There were also significant disparities by income level. 2019-2020 data show the largest disparity gap was between those at 400+ percent poverty threshold and those <100 percent of the poverty threshold with the former experiencing a higher rate of flu vaccine. Recent 2022-2023 data show that this disparity persists and the rate of vaccinations among those 400+ percent poverty threshold slightly increased while the rate among individuals at or less than 100 percent of the poverty threshold decreased. The absolute disparity gap has increased from a maximal rate difference of 11.3 vaccinations to 17.1 vaccinations, and the relative disparity gap has slightly increased from a maximal rate ratio of 1.25 to 1.41

2018-2019 data show metropolitan areas had a slightly higher flu vaccination rate compared to non-metropolitan areas. Recent 2022-2023 data show the flu vaccination rate for metropolitan areas remaining stagnant while the rate has dropped in non-metropolitan areas. The absolute disparity gap increased from a maximal rate difference of 1.8 vaccinations to 7.5 vaccinations and the relative disparity gap worsened from a maximal rate ratio of 1.0 to 1.2.

*Maternal and Infant Health*

MICH-04

Baseline data from 2018 data show the largest maternal health gap due to race was between Black or African American only individuals compared to Hispanic or Latino individuals with the former experiencing a higher rate of maternal deaths — a maximal rate difference of 25.5 deaths and maximal rate ratio of 3.2 Recent 2023 data show the largest disparity gap is now between Black or African American only individuals and Asian only individuals, with black individuals still experiencing a disproportionate number of maternal deaths. The maximal rate difference has risen to 39.6 deaths, and the maximal rate ratio has risen to 4.7.

When stratified by geographic location, baseline data from 2018 data show metropolitan areas experience a lower rate of maternal deaths compared to non-metropolitan areas. Recent 2023 data show slight jumps in rates of maternal deaths in both locations with the absolute disparity gap and relative disparity gap staying relatively stagnant with a rate difference of 4.6 and rate ratio of 1.26.

2018 data show the largest age disparity gap was between individuals aged 35+ and those aged 20-24 with the former experiencing a higher rate of maternal deaths. The latest data from 2023 show a decrease in maternal death rate for those 35+ and a slight increase in maternal death rate for those 2024. Since 2017, The absolute and relative disparity have narrowed from a rate difference of 30.6 to 20 and a rate ratio from 3.97 to 2.74.

**Appendix Tables**

**Appendix Table A1: Measurement Methods for LHIs**

| Leading Health Indicators (LHIs) | Sample Criteria | Data Source |
| --- | --- | --- |
| AHS-01 | Percent of persons under 65 years who had medical insurance in 2019 | National Health Interview Survey |
| SDOH--02 | Percent of working age population aged 16 to 64 years who were employed in 2018 | Current Population Survey Annual Social and Economic Supplement Census |
| NWS-01 | Percent of households who were food insecure in 2018 | Current Population Survey Food Security Supplement |
| AH-05 | Percent of those attending public and private schools who had reading skills at or above proficient achievement level for their grade in 2017 | National Assessment of Educational Progress |
| MHMD-01 | Number of suicides per 100,000 population in 2018 | National Vital Statistics System |
| TU-02 | Percent of adults aged 18+ who were current cigarette smokers in 2019 | National Health Interview Survey |
| TU-04 | Percent of students in grades 6 through 12 who used cigarettes, e-cigarettes, cigars, smokeless tobacco, hookah, pipe tobacco, and/or bidis in the past 30 days in 2018 | National Youth Tobacco Survey |
| SU-10 | Percent of persons aged 21+ who reported binge drinking in the past 30 days in 2018 | National Survey on Drug Use and Health |
| SU-03 | Number of drug overdose deaths per 100,000 population that occurred in 2018 | National Vital Statistics System |
| IVP-09 | Number of homicides per 100,000 population that occurred in 2018 | National Vital Statistics System |
| MHMD-06 | Percent of adolescents aged 12 to 17 years with MDEs who received treatment in the past 12 months, as reported in 2021 | National Survey on Drug Use and Health |
| OH-08 | Percent of children, adolescents, and adults who used the oral health care system in 2016 | Medical Expenditure Panel |
| HIV-02 | Percent of persons aged 13+ living with HIV who were aware of their HIV infection in 2017 | National HIV Surveillance System |
| IID-09 | Percent of persons aged 6 months and over who were vaccinated against seasonal influenza for the flu season 2019-20 | National Health Interview Survey |
| HDS-05 | Percent of adults aged 18+ with high blood pressure/hypertension who had it under control in 2017-20 | National Health and Nutrition Examination Survey |
| D-01 | Number of new cases of diabetes per 1000 adults aged 18 to 84 years in past 12 months reported in 2019-21 | National Health Interview Survey |
| PA-05 | Percent of adults aged 18+ who met the guidelines for aerobic physical activity and muscle strengthening activity during their leisure time in 2020 | National Health Interview Survey |
| NWS-04 | Percent of children and adolescents aged 2 to 19 years who had obesity in 2013-16 | National Health and Nutrition Examination Survey |
| NWS-10 | Percent was the mean percentage of calories from added sugars consumed by persons aged 2 years and over in 2013-16 | National Health and Nutrition Examination Survey |
| C-07 | Percent of adults aged 45 to 75 years who received a colorectal cancer screening based on the most recent guidelines in 2023 | National Health Interview Survey |
| EH-01 | Air Quality Index (AQI) - weighted people days exceeded 100 on the AQI in 2016-18 | EPA Air Quality System repository |
| MICH-04 | Maternal deaths per 100,000 live births that occurred in 2018 | National Vital Statistics System |
| MICH-02 | Infant deaths per 1000 live births that occurred within the first year of life in 2017 | Linked Birth/Infant Death Data Set |

*^a^ Data Source*: *Healthy People 2030,* *Office of Disease Prevention and Health Promotion*, *U.S. Department of Health and Human Services*

**Appendix Table A2: Progress towards Equitable Access and Social Determinants of Health (SDOH)**

| Equitable Access and Social Determinants of Health | Baseline Value | Current Value | Target Value | Direction-Corrected Percent Change from Baseline (PCB) | Percent of Targeted Change Achieved (PTCA) |
| --- | --- | --- | --- | --- | --- |
| Increase the proportion of people with health insurance (AHS‑01)^*^ | 88 (2019) | 91.1 (2023) | 92.4 | 3.5 | 70.5 |
| Increase employment in working-age people (SDOH‑02)^*^ | 70.6 (2018) | 71.5 (2023) | 75 | 1.3 | 20.5 |
| Reduce household food insecurity and hunger (NWS‑01)* | 11.1 (2018) | 13.5 (2023) | 6 | -21.6 | N/A |
| Increase the proportion of 4th-graders with proficient reading skills (AH‑05)^*^ | 36.6 (2017) | 31.1 (2024) | 41.5 | -15.0 | N/A |

*^a^ Leading Health Indicators (LHIs) with only baseline values available=^*

*^b^ Changes were significant = **

*^c^ Data Source*: *Healthy People 2030,* *Office of Disease Prevention and Health Promotion*, *U.S. Department of Health and Human Services*

*^e^ Target were established by federal subject-matter experts (SMEs*) convened in HP2030 workgroups that include statisticians and epidemiologists from federal agencies.^3^

*^f^ Normalized progress of each LHI used the following formula: (Current - Baseline)/(Target - Baseline) x 100%*

**Appendix Table A3: AH-05 Disparity Data**

| Year | Stratifier | Higher group (value; CI; SE) | Lower group (value; CI; SE) |
| --- | --- | --- | --- |
| 2017 | Race/ethnicity | Asian only, not Hispanic or Latino (58.7; CI: 55.8–61.6; SE: 1.45) | Black or African American only, not Hispanic (19.9; CI: 18.8–21; SE: 0.547) |
| 2017 | School lunch eligibility | Not eligible (51.6; CI: 50.8–52.5; SE: 0.441) | Eligible (21.8; CI: 21.2–22.5; SE: 0.312) |
| 2022 | School lunch eligibility | Not eligible (46.4; CI: 45.3–47.5; SE: 0.553) | Eligible (19.3; CI: 18.4–20.2; SE: 0.469) |
| 2024 | Race/ethnicity | Asian only, not Hispanic or Latino (53; CI: 49.6–56.5; SE: 4.87) | American Indian/Alaska Native only, not Hispanic (13.9; CI: 9.8–19.4; SE: 2.16) |

| Difference (Higher − Lower) | Diff SE | Diff LCL | Diff UCL | Diff Sig? | Ratio (Higher ÷ Lower) | SE ln(Ratio) | Ratio LCL | Ratio UCL | Ratio Sig? | Units |
| --- | --- | --- | --- | --- | --- | --- | --- | --- | --- | --- |
| 38.8 | 1.550 | 35.8 | 41.8 | Yes | 3.0 | 0.037 | 2.7 | 3.2 | Yes | % |
| 29.8 | 0.540 | 28.7 | 30.9 | Yes | 2.4 | 0.017 | 2.3 | 2.4 | Yes | % |
| 27.1 | 0.725 | 25.7 | 28.5 | Yes | 2.4 | 0.027 | 2.3 | 2.5 | Yes | % |
| 39.1 | 5.326 | 28.7 | 49.5 | Yes | 3.8 | 0.181 | 2.7 | 5.4 | Yes | % |

**Appendix Table A4: NWS-01 Disparity Data**

| Year | Stratifier | Higher group (value; CI; SE) | Lower group (value; CI; SE) |
| --- | --- | --- | --- |
| 2018 | Disability status | One or more adults with disabilities (21.1; CI: 20–22.1; SE: 0.537) | No adults with disabilities (8.1; CI: 7.7–8.5; SE: 0.21) |
| 2018 | Family income | <100% FPL (35.3; CI: 33.3–37.3; SE: 1.02) | ≥400% FPL (1.9; CI: 1.6–2.2; SE: 0.144) |
| 2023 | Disability status | One or more adults with disabilities (23.2; CI: 22.1–24.3; SE: 0.544) | No adults with disabilities (10.5; CI: 10–11.1; SE: 0.276) |
| 2023 | Family income | <100% FPL (38.6; CI: 36.6–40.8; SE: 1.06) | ≥400% FPL (3.5; CI: 3.1–4; SE: 0.22) |

| Difference (Higher − Lower) | Diff SE | Diff LCL | Diff UCL | Diff Sig? | Ratio (Higher ÷ Lower) | SE ln(Ratio) | Ratio LCL | Ratio UCL | Ratio Sig? | Units |
| --- | --- | --- | --- | --- | --- | --- | --- | --- | --- | --- |
| 13.0 | 0.577 | 11.9 | 14.1 | Yes | 2.6 | 0.036 | 2.4 | 2.8 | Yes | % |
| 33.4 | 1.027 | 31.4 | 35.4 | Yes | 18.6 | 0.081 | 15.8 | 21.8 | Yes | % |
| 12.7 | 0.610 | 11.5 | 13.9 | Yes | 2.2 | 0.035 | 2.1 | 2.4 | Yes | % |
| 35.1 | 1.087 | 33.0 | 37.2 | Yes | 11.0 | 0.069 | 9.6 | 12.6 | Yes | % |

**Appendix Table A5: Progress towards Behavioral Health Risks and Harm**

| Behavioral Health Risk and Harm | Baseline Value | Current Value | Target Value | Direction-Corrected Percent Change from Baseline (PCB) | Percent of Targeted Change Achieved (PTCA) |
| --- | --- | --- | --- | --- | --- |
| Increase the proportion of adolescents with depression who get treatment — (MHMD‑06)* | 40 (2021) | 50.7 (2023) | 44.9 | 26.8 | 218.4 |
| Reduce binge drinking in people aged 21 and over (SU‑10) | 23.8 (2021) | 22.0 (2024) | 22.7 | 7.6 | 163.6 |
| Reduce current cigarette smoking in adults (TU‑02)* | 14.2 (2019) | 11.0 (2023) | 6.1 | 22.5 | 39.5 |
| Reduce the suicide rate (MHMD‑01) | 14.2 (2018) | 14.1 (2023) | 12.8 | 0.7 | N/A |
| Reduce drug overdose deaths (SU‑03)* | 20.7 (2018) | 31.3 (2023) | 20.7 | -51.2 | N/A |
| Reduce current tobacco use in adolescents (TU‑04)* | 18.3 (2018) | 10.0 (2023) | 11.3 | 45.4 | 118.6 |
| Reduce homicides (IVP‑09)* | 5.9 (2018) | 7.1 (2023) | 5.5 | -20.3 | N/A |

*^a^ Leading Health Indicators (LHIs) with only baseline values available=^*

*^b^ Changes were significant = **

*^c^ Data Source*: *Healthy People 2030,* *Office of Disease Prevention and Health Promotion*, *U.S. Department of Health and Human Services*

*^e^ Target were established by federal subject-matter experts (SMEs*) convened in HP2030 workgroups that include statisticians and epidemiologists from federal agencies.^3^

*^f^ Normalized progress of each LHI used the following formula: (Current - Baseline)/(Target - Baseline) x 100%*

**Appendix Table A6: IVP-09 Disparity Data**

| Year | Stratifier | Higher group (value; CI; SE) | Lower group (value; CI; SE) |
| --- | --- | --- | --- |
| 2018 | Age group | 18–44 years (10.6; SE: 0.095) | 65+ years (2.2; SE: 0.065) |
| 2018 | Geography | Metropolitan (6; SE: 0.047) | Non-metropolitan (5.5; SE: 0.117) |
| 2018 | Sex | Male (9.3; SE: 0.078) | Female (2.5; SE: 0.04) |
| 2021 | Geography | Metropolitan (8.3; SE: 0.055) | Non-metropolitan (7.1; SE: 0.134) |
| 2023 | Age group | 18–44 years (12.3; SE: 0.101) | 65+ years (2.3; SE: 0.062) |
| 2023 | Sex | Male (11.3; SE: 0.085) | Female (2.8; SE: 0.042) |

| Difference (Higher − Lower) | Diff SE | Diff LCL | Diff UCL | Diff Sig? | Ratio (Higher ÷ Lower) | SE ln(Ratio) | Ratio LCL | Ratio UCL | Ratio Sig? | Units |
| --- | --- | --- | --- | --- | --- | --- | --- | --- | --- | --- |
| 8.4 | 0.115 | 8.2 | 8.6 | Yes | 4.8 | 0.031 | 4.5 | 5.1 | Yes | deaths per 100,000 |
| 0.5 | 0.126 | 0.3 | 0.7 | Yes | 1.1 | 0.023 | 1.0 | 1.1 | Yes | deaths per 100,000 |
| 6.8 | 0.088 | 6.6 | 7.0 | Yes | 3.7 | 0.018 | 3.6 | 3.9 | Yes | deaths per 100,000 |
| 1.2 | 0.145 | 0.9 | 1.5 | Yes | 1.2 | 0.020 | 1.1 | 1.2 | Yes | deaths per 100,000 |
| 10.0 | 0.119 | 9.8 | 10.2 | Yes | 5.3 | 0.028 | 5.1 | 5.7 | Yes | deaths per 100,000 |
| 8.5 | 0.095 | 8.3 | 8.7 | Yes | 4.0 | 0.017 | 3.9 | 4.2 | Yes | deaths per 100,000 |

**Appendix Table A7: SU-03 Disparity Data**

| Year | Stratifier | Higher group (value; CI; SE) | Lower group (value; CI; SE) |
| --- | --- | --- | --- |
| 2018 | Age group | 45–64 years (31.7; SE: 0.195) | <18 years (0.5; SE: 0.025) |
| 2018 | Geography | Metropolitan (21; SE: 0.088) | Non-metropolitan (19; SE: 0.218) |
| 2018 | Sex | Male (27.9; SE: 0.134) | Female (13.6; SE: 0.093) |
| 2021 | Geography | Metropolitan (32.4; SE: 0.108) | Non-metropolitan (32.2; SE: 0.285) |
| 2023 | Age group | 45–64 years (51.2; SE: 0.249) | <18 years (1.4; SE: 0.043) |
| 2023 | Sex | Male (44.3; SE: 0.166) | Female (18.3; SE: 0.107) |

| Difference (Higher − Lower) | Diff SE | Diff LCL | Diff UCL | Diff Sig? | Ratio (Higher ÷ Lower) | SE ln(Ratio) | Ratio LCL | Ratio UCL | Ratio Sig? | Units |
| --- | --- | --- | --- | --- | --- | --- | --- | --- | --- | --- |
| 31.2 | 0.197 | 30.8 | 31.6 | Yes | 63.4 | 0.050 | 57.4 | 70.0 | Yes | deaths per 100,000 |
| 2.0 | 0.235 | 1.5 | 2.5 | Yes | 1.1 | 0.012 | 1.1 | 1.1 | Yes | deaths per 100,000 |
| 14.3 | 0.163 | 14.0 | 14.6 | Yes | 2.1 | 0.008 | 2.0 | 2.1 | Yes | deaths per 100,000 |
| 0.2 | 0.305 | -0.4 | 0.8 | No | 1.0 | 0.009 | 1.0 | 1.0 | No | deaths per 100,000 |
| 49.8 | 0.253 | 49.3 | 50.3 | Yes | 36.6 | 0.031 | 34.4 | 38.9 | Yes | deaths per 100,000 |
| 26.0 | 0.197 | 25.6 | 26.4 | Yes | 2.4 | 0.007 | 2.4 | 2.5 | Yes | deaths per 100,000 |

**Appendix Table A8: MHMD-01 Disparity Data**

| Year | Stratifier | Higher group (value; CI; SE) | Lower group (value; CI; SE) |
| --- | --- | --- | --- |
| 2018 | Age group | 45–64 years (20.1; SE: 0.155) | <18 years (2.5; SE: 0.058) |
| 2018 | Sex | Male (22.8; SE: 0.119) | Female (6.2; SE: 0.062) |
| 2023 | Age group | 45–64 years (18.8; SE: 0.151) | <18 years (2.2; SE: 0.055) |
| 2023 | Sex | Male (22.7; SE: 0.117) | Female (5.9; SE: 0.059) |

| Difference (Higher − Lower) | Diff SE | Diff LCL | Diff UCL | Diff Sig? | Ratio (Higher ÷ Lower) | SE ln(Ratio) | Ratio LCL | Ratio UCL | Ratio Sig? | Units |
| --- | --- | --- | --- | --- | --- | --- | --- | --- | --- | --- |
| 17.6 | 0.165 | 17.3 | 17.9 | Yes | 8.0 | 0.024 | 7.7 | 8.4 | Yes | deaths per 100,000 |
| 16.6 | 0.134 | 16.3 | 16.9 | Yes | 3.7 | 0.011 | 3.6 | 3.8 | Yes | deaths per 100,000 |
| 16.6 | 0.161 | 16.3 | 16.9 | Yes | 8.5 | 0.026 | 8.1 | 9.0 | Yes | deaths per 100,000 |
| 16.8 | 0.131 | 16.5 | 17.1 | Yes | 3.8 | 0.011 | 3.8 | 3.9 | Yes | deaths per 100,000 |

**Appendix Table A9: Progress towards Preventive Health and Chronic Disease Management**

| Preventive Health and Chronic Disease Management | Baseline Value | Current Value | Target Value | Direction-Corrected Percent Change from Baseline (PCB) | Percent of Targeted Change Achieved (PTCA) |
| --- | --- | --- | --- | --- | --- |
| Increase use of the oral health care system (OH‑08)* | 43.2 (2016) | 45.5 (2022) | 45 | 5.3 | 127.8 |
| Increase knowledge of HIV status (HIV‑02)* | 85.8 (2017) | 87.2 (2022) | 95 | 1.6 | 15.2 |
| Reduce consumption of added sugars by people aged 2 and over (NWS-10) | 13.5 (2013-16) | 13.2 (2017-20) | 11.5 | 2.2 | N/A |
| Increase physical activity in adults (PA-05) | 25.2 (2020) | 26.4 (2024) | 29.7 | 4.8 | 26.7 |
| Increase control of high blood pressure in adults (HDS‑05)^ | 16.1 (2017-20) | 20.7 (2021-2023) | 18.9 | 28.6 | 164.3 |
| Reduce diabetes cases diagnosed yearly (D‑01)^ | 5.5 (2019-21) | N/A | 4.8 | NA | N/A |
| Increase the proportion of adults who get screened for colorectal cancer — (C‑07)^ | 63.5 (2023) | N/A | 72.8 | NA | N/A |
| Increase the proportion of people who get the flu vaccine every year (IID09) | 51.6 (2019-20) | 50.5 (2022-23) | 70 | -2.1 | N/A |
| Reduce exposure to unhealthy air (EH01) | 4,295,962,018 (2016-18) | 4,534,737,587 (2019-2021) | 3,866,365,816 | -5.6 | N/A |
| Reduce childhood and adolescent obesity (NWS04) | 17.8 (2013-16) | 21.1 (2021-2023) | 15.5 | -18.5 | N/A |

*^a^ Leading Health Indicators (LHIs) with only baseline values available =^*

*^b^ Changes were significant = **

*^c^ Data Source*: *Healthy People 2030,* *Office of Disease Prevention and Health Promotion*, *U.S. Department of Health and Human Services*

*^e^ Target were established by federal subject-matter experts (SMEs*) convened in HP2030 workgroups that include statisticians and epidemiologists from federal agencies.^3^

*^f^ Normalized progress of each LHI used the following formula: (Current - Baseline)/(Target - Baseline) x 100%*

**Appendix Table A10: IID-09 Disparity Data**

| Year | Stratifier | Higher group (value; CI; SE) | Lower group (value; CI; SE) |
| --- | --- | --- | --- |
| 2018 | Geography | Metropolitan (51.9; CI: 50.4–52.8; SE: 0.595) | Non-metropolitan (50.1; CI: 47.5–52.8; SE: 1.34) |
| 2019 | Family income | ≥400% FPL (57.4; CI: 55.7–59; SE: 0.836) | <100% FPL (46.1; CI: 41.9–50.4; SE: 2.18) |
| 2019 | Insurance status | Public insured (56; CI: 53.7–58.3; SE: 1.16) | Uninsured (23.9; CI: 21–27.2; SE: 1.58) |
| 2022 | Family income | ≥400% FPL (58.5; CI: 57.1–60; SE: 0.755) | <100% FPL (41.4; CI: 38.2–44.8; SE: 1.7) |
| 2022 | Geography | Metropolitan (51.5; CI: 50.3–52.8; SE: 0.639) | Non-metropolitan (44; CI: 41.3–46.8; SE: 1.39) |
| 2022 | Insurance status | Private insured (53.4; CI: 52.1–54.8; SE: 0.698) | Uninsured (22.3; CI: 19.5–25.6; SE: 1.55) |

| Year | Stratifier | Higher group (value; CI; SE) | Lower group (value; CI; SE) |
| --- | --- | --- | --- |
| 2018 | Geography | Metropolitan (51.9; CI: 50.4–52.8; SE: 0.595) | Non-metropolitan (50.1; CI: 47.5–52.8; SE: 1.34) |
| 2019 | Family income | ≥400% FPL (57.4; CI: 55.7–59; SE: 0.836) | <100% FPL (46.1; CI: 41.9–50.4; SE: 2.18) |
| 2019 | Insurance status | Public insured (56; CI: 53.7–58.3; SE: 1.16) | Uninsured (23.9; CI: 21–27.2; SE: 1.58) |
| 2022 | Family income | ≥400% FPL (58.5; CI: 57.1–60; SE: 0.755) | <100% FPL (41.4; CI: 38.2–44.8; SE: 1.7) |
| 2022 | Geography | Metropolitan (51.5; CI: 50.3–52.8; SE: 0.639) | Non-metropolitan (44; CI: 41.3–46.8; SE: 1.39) |
| 2022 | Insurance status | Private insured (53.4; CI: 52.1–54.8; SE: 0.698) | Uninsured (22.3; CI: 19.5–25.6; SE: 1.55) |

**Appendix Table A11: Progress towards Maternal and Infant Health**

| Maternal and Infant Health | Baseline Value | Current Value | Target Value | Direction-Corrected Percent Change from Baseline (PCB) | Percent of Targeted Change Achieved (PTCA) |
| --- | --- | --- | --- | --- | --- |
| Reduce the rate of infant deaths (MICH‑02)^*^ | 5.8 (2017) | 5.61 (2022) | 5.0 | 3.3 | 23.8 |
| Reduce maternal deaths (MICH‑04)^*^ | 17.4 (2018) | 18.6 (2023) | 15.7 | -6.9 | N/A |

*^a^ Leading Health Indicators (LHIs) with only baseline values available =^*

*^b^ Changes were significant = **

*^c^ Data Source*: *Healthy People 2030,* *Office of Disease Prevention and Health Promotion*, *U.S. Department of Health and Human Services*

*^e^ Target were established by federal subject-matter experts (SMEs*) convened in HP2030 workgroups that include statisticians and epidemiologists from federal agencies.^3^

*^f^ Normalized progress of each LHI used the following formula: (Current - Baseline)/(Target - Baseline) x 100%*

**Appendix Table A12: MICH-04 Disparity Data**

| Year | Stratifier | Higher group (value; CI; SE) | Lower group (value; CI; SE) |
| --- | --- | --- | --- |
| 2018 | Age group | 35+ years (40.9; SE: 2.43) | 20–24 years (10.3; SE: 1.19) |
| 2018 | Geography | Non-metropolitan (21.6; SE: 2.05) | Metropolitan (16.7; SE: 0.713) |
| 2018 | Race/ethnicity | Black or African American only, not Hispanic (37.3; SE: 2.6) | Hispanic or Latino (11.8; SE: 1.16) |
| 2023 | Age group | 35+ years (31.5; SE: 2.05) | 20–24 years (11.5; SE: 1.37) |
| 2023 | Geography | Non-metropolitan (22.6) | Metropolitan (18) |
| 2023 | Race/ethnicity | Black or African American only, not Hispanic (50.3; SE: 3.2) | Asian only, not Hispanic or Latino (10.7; SE: 2.22) |

| Difference (Higher − Lower) | Diff SE | Diff LCL | Diff UCL | Diff Sig? | Ratio (Higher ÷ Lower) | SE ln(Ratio) | Ratio LCL | Ratio UCL | Ratio Sig? | Units |
| --- | --- | --- | --- | --- | --- | --- | --- | --- | --- | --- |
| 30.6 | 2.707 | 25.3 | 35.9 | Yes | 4.0 | 0.130 | 3.1 | 5.1 | Yes | deaths per 100,000 live births |
| 4.9 | 2.173 | 0.6 | 9.2 | Yes | 1.3 | 0.104 | 1.1 | 1.6 | Yes | deaths per 100,000 live births |
| 25.5 | 2.845 | 19.9 | 31.1 | Yes | 3.2 | 0.120 | 2.5 | 4.0 | Yes | deaths per 100,000 live births |
| 20.0 | 2.462 | 15.2 | 24.8 | Yes | 2.7 | 0.135 | 2.1 | 3.6 | Yes | deaths per 100,000 live births |
| 4.6 |  |  |  |  | 1.3 |  |  |  |  | deaths per 100,000 live births |
| 39.6 | 3.895 | 32.0 | 47.2 | Yes | 4.7 | 0.217 | 3.1 | 7.2 | Yes | deaths per 100,000 live births |
